# Genetic mapping of adult-plant resistance genes to powdery mildew in triticale

**DOI:** 10.1007/s13353-021-00664-x

**Published:** 2021-09-24

**Authors:** Mateusz Dyda, Mirosław Tyrka, Gabriela Gołębiowska, Marcin Rapacz, Maria Wędzony

**Affiliations:** 1 Chair of Genetics, Institute of Biology, Pedagogical University of Cracow, Podchorążych 2, 30-084 Kraków, Poland; 2 Department of Biotechnology and Bioinformatics, Faculty of Chemistry, Rzeszów University of Technology, Rzeszów, Poland; 3 Department of Plant Breeding, Physiology and Seed Science, University of Agriculture in Kraków, Podłużna 3, 30-239 Krakow, Poland

**Keywords:** Triticale, Powdery mildew, Genetic map, Quantitative trait locus, Candidate genes

## Abstract

Triticale is a cereal of high economic importance; however, along with the increase in the area of this cereal, it is more often infected by the fungal pathogen *Blumeria graminis*, which causes powdery mildew. The rapid development of molecular biology techniques, in particular methods based on molecular markers may be an important tool used in modern plant breeding. Development of genetic maps, location of the QTLs defining the region of the genome associated with resistance and selection of markers linked to particular trait can be used to select resistant genotypes as well as to pyramidize several resistance genes in one variety. In this paper, we present a new, high-density genetic map of triticale doubled haploids (DH) population “Grenado” × “Zorro” composed of DArT, silicoDArT, and SNP markers. Composite interval mapping method was used to detect eight QTL regions associated with the area under disease progress curve (AUDPC) and 15 regions with the average value of powdery mildew infection (avPM) based on observation conducted in 3-year period in three different locations across the Poland. Two regions on rye chromosome 4R, and single *loci* on 5R and 6R were reported for the first time as regions associated with powdery mildew resistance. Among all QTLs, 14 candidate genes were identified coded cyclin-dependent kinase, serine/threonine-protein kinase-like protein as well as AMEIOTIC 1 homolog DYAD-like protein, DETOXIFICATION 16-like protein, and putative disease resistance protein RGA3. Three of identified candidate genes were found among newly described QTL regions associated with powdery mildew resistance in triticale.

**Supplementary Information:**

The online version contains supplementary material available at 10.1007/s13353-021-00664-x.

## Introduction

Triticale (x*Triticosecale* Wittm.) is a human-made wheat-rye hybrid commercialized in the late 1960s (Ammar et al. 2004). Currently cultivated, hexaploid triticale (2*n* = 6x = 42, AABBRR) accumulates important traits determined by wheat (A and B) and rye (R) genomes (Walker et al. 2011; Klocke et al. 2013). In the last years, triticale has raised its economic importance mainly in Europe. Poland with triticale cultivation area of 1.3 million ha contribute to 1/3 of world production and remains the top producer of this crop (Faostat 2020). Simultaneously, risk of infection by the biotrophic fungal pathogen *Blumeria graminis* (DC.) Speer which causes powdery mildew has recently increased.

The epidemic appearance of powdery mildew on triticale has been observed in several European countries, including Belgium, France, Germany, and Poland as well (Walker et al. 2011). An epidemics of powdery mildew causes yield drop and requires preventive use of fungicides. The cultivation of triticale varieties resistant to pathogenic fungi offers the most economical and environmentally friendly alternative to chemical protection. So far, 50 loci with more than 78 genes/alleles associated with powdery mildew resistance have been identified on 18 chromosomes of bread wheat and its relatives (Yang et al. 2017) and only 8 resistance genes have been identified in rye (Tyrka and Chelkowski 2004; Schlegel and Korzun, 2021). Many of these resistance genes were broken down by the new races of *B. graminis* (Menardo et al. 2016), and triticale can benefit both from genes present in rye and introduced into wheat from alien species (Tyrka and Chelkowski 2004; Alam et al. 2013; Schlegel and Korzun, 2021).

Techniques based on DNA molecular markers has become an indispensable tool in modern plant breeding used to monitor introgression and for accumulation of desired genes in breeding materials (Yang et al. 2015). A number of methods based on DNA hybridization (Jaccoud et al. 2001; Cavanagh et al. 2013; Jordan et al. 2015) and next generation sequencing (Vikram et al. 2016; Riaz et al. 2016; Baloch et al. 2017) have been developed and used for wheat or triticale genotyping. Recently, sequencing efforts resulted in assembling of wheat and rye genome (IWGSC 2014, 2018; Bauer et al. 2017; Rabanus-Wallaceet al. 2021). However, in species with sequenced genomes, genetic maps are useful for detecting chromosomal rearrangements (Wingen et al. 2017) and necessary for quantitative trait loci (QTLs) localization (Vinod 2009; Holtz et al. 2016). Therefore, a number of genetic maps have already been developed for wheat (Somers et al. 2004; Mantovani et al. 2008), rye (Korzun et al. 2001; Milczarski et al. 2011), and triticale (Alheit et al. 2011; Tyrka et al. 2011, 2015; Karbarz et al. 2020; Wąsek et al. 2021).

The aims of this study were to (1) develop a high-density genetic map for hexaploid winter triticale composed of diversity arrays technology (DArT), silicoDArT, and DArT-based single nucleotide polymorphism (SNP) markers using DH population of lines derived from two triticale cultivars and (2) identify QTL regions and candidate genes responsible for an adult-plant resistance of triticale (x*Triticosecale* Wittm.) to powdery mildew infection in natural field conditions.

## Materials and methods

### Experimental population

The mapping population used in this study consisted 168 doubled haploid (DH) lines derived from F_1_ hybrid “Grenado” × “Zorro.” “Grenado” was resistant parent and “Zorro” was highly susceptible to infection of *B. graminis*. These cultivars were registered by Strzelce Plant Breeders Ltd (Plant Breeding and Acclimatization Institute Group, Poland) and Danko Plant Breeders Ltd, respectively. The DH lines were obtained at the Department of Cell Biology of Institute of Plant Physiology Polish Academy of Science (IPP PAS) in Kraków by the anther culture method according to Wędzony (2003).

### Plant growth conditions and phenotyping

For the first year of field experiment, lines were reproduced in greenhouse and healthy leaves were sampled for DNA isolation. Seeds of parental lines and each DH line were germinated in plastic pots (3.7 dm^3^; nine seeds per pot), previously filled with a homogeneous mixture of sand and soil (3:1; v/v). The pots were placed for 8 weeks in a cool chamber at 4 °C (± 1 °C), photoperiod 10-h light/14-h dark. Next, the plants were transferred into a greenhouse chambers with air temperature 26–28/18 °C (± 2 °C) day/night and relative air humidity 40%. All plants were irrigated once a week with a Hoagland’s solution (Hoagland 1948). The seeds were obtained from individual DH lines and their parents from bagged spikes in the greenhouse in the IPP PAS in Kraków. Seed material for the second and the third year of experiment was obtained in field conditions in Danko Plant Breeders Ltd by isolation of five spikes per each DH line before flowering.

Powdery mildew (PM) resistance was assessed in field conditions for three years (2013–2015) in three localizations spread across Poland: Choryń (52° 2′ 26″ N 16° 46′ 59″ E; all three seasons), Laski (51° 47′ N 21° 12′ E; season 2012/2013 and 2013/2014) and Modzurów (50° 9′ 20″ N 18° 7′ 52″ E; season 2014/2015). The lines were sown in two 1-m long rows at the 20 × 2.5 cm spacing. Susceptible cultivar “Zorro” was sewed as spreader every 20 plots. The chemical protection was not applied during plant growth and powdery mildew infection was measured under natural infection. Disease was assessed on a whole plot basis using a 0–9 scale (McNeal et al. 1971), where 0 is immune and 9 is very susceptible (Ziems et al. 2014). Observations were made in periods of heading, flowering, and seed formation. Depending on the weather conditions during field experiments (high temperature and drought) which led to death of some plants, field observations of the PM degree were conducted in one, two, or three stages. Data which were recorded 3 times during one vegetative season in Choryń were used to calculate area under disease progress curve (AUDPC) (Shaner and Finney 1977; Finckh et al. 1999; Jeger and Viljanen-Rollinson 2001), whereas data recorded once or 2 times were used to determine the average value of powdery mildew infection (avPM) according to the 9-grade scale.

### DNA isolation and genotyping

Genomic DNA was isolated from a 90- to100-mg sample of two leaves per each DH line and both parents. The samples were frozen in liquid nitrogen and stored at − 60 °C until the isolation was made. Total genomic DNA isolation for each sample was carried out using the GeneJET Plant Genomic DNA Purification Mini Kit (Thermo Scientific, Waltham, USA). The concentration and purity of the DNA was evaluated using a UV–Vis Q500 (Quawell, San Jose, USA) spectrophotometer. DNA was sent to Diversity Arrays Technology (Yarralumla, Australia) both for profiling using triticale high -resolution array (DArT) with probes representing markers from rye, wheat, and triticale (*rPt*, *wPt*, and *tPt*, respectively) and for DArTseq analysis.

### Construction of the genetic map

De novo mapping approach was used to construct genetic map for “Grenado” × “Zorro” DH population. Markers of unknown parental origin and present the frequency < 5% and > 95% were removed from the dataset. All types of DArT markers were binned with QTL IciMapping (Wang et al. 2016). Segregation data were analyzed using JoinMap4 (Van Ooijen 2006) to group all markers using the logarithm of odds (LOD) > 3. Markers within these groups were recurrently ordered using the maximum likelihood option of JoinMap and the RECORD program (Van Os et al. 2005). To establish the marker order, all linkage groups identified for “Grenado” × “Zorro” DH population were compared to reference genetic maps of triticale (Tyrka et al. 2015), reference genome of wheat at URGI (https://urgi.versailles.inra.fr) and partial rye genome (Bauer et al. 2017).

### Statistical, QTL, and candidate genes analysis

Mean values from all observations were used to calculate the Pearson’s correlations. The Shapiro–Wilk test was performed to assess deviations from a normal distribution as well as skewness and kurtosis were calculated using Statistica version 12.0 (StatSoft, Inc. USA). High-density genetic map and complete phenotyping data of the degree of powdery mildew infection intriticale were exploited in QTL analysis using WinQTLCartographer2.5 software (Wang et al. 2012). Composite interval mapping (CIM) analysis with a 1000-permutation test and walk speed of 1.0 cM were performed to declare a significant QTL. The LOD threshold was between 2.1 and 8.3 depending on the trait. The percentage of the phenotypic variation covered by QTL was calculated with a single factor regression (*R*^2^) and the favorable alleles in each QTL region were selected, based on the additive (Add) effect (negative additive effect refers to cv. “Zorro” while positive to cv. “Grenado”). Candidate genes analysis was performed according to method detailed described by Wąsek et al. (2021). 

## Results

### Phenotypic analysis

Phenotypic variation in powdery mildew infection was assessed for all lines of the “Grenado” × “Zorro” DH population and for both parental lines in Choryń, Laski, and Modzurów during all three vegetative seasons (Table 1, Fig. S1). According to Shapiro–Wilk test, distributions of AUDPC and avPM values over locations and seasons not deviated significantly from a normal distribution. Skewness and kurtosis values also confirmed the proper distribution of observations for the experiments (Table 1). AUDPC values varied significantly depending on the year of experiment. Although, maximum values of AUDPC between years were similar and amounted to 2675.6 and 2530.6, different dynamics of disease development was observed and minimum AUDPC values ranged from 65.2 and 1678.3 in 2015 and 2013, respectively. Average avPM values ranged from 3.3 to 5.7 (Table 1). Besides, statistically significant highly positive correlations between different powdery mildew scores were found within locations that reflect disease progression. Powdery mildew distribution for Choryń in 2015 was significantly, positively correlated also with observations in Modzurów and Laski (Table 2).

**Table 1:** The values range of powdery mildew resistance measured in 9-grade scale for all 168 DH lines of “Grenado” × “Zorro” mapping population evaluated in all localizations in three years, mean value and standard deviation, the normality test using Shapiro–Wilk statistics as well as skewness and kurtosis values

Exp. location	Exp. season	Exp. term	Trait	Minimum–maximum	Mean value ± SD	Normality	Skewness	Kurtosis
**Choryń**	2013	1	AUDPC	1678.3–2675.6	2196.1 ± 185.6	0.98	− 0.5532	− 0.1012
		2				0.96	0.6779	0.0929
		3				0.85	0.7487	− 0.0256
	2014	1	avPM	2.0–8.0	5.6 ± 1.2	0.89	− 0.3021	0.0314
	2015	1	AUDPC			0.97	− 0.5442	0.7658
		2		65.2–2530.6	760.6 ± 481.1	0.96	− 0.3177	0.2544
		3				0.98	0.1285	0.8870
**Laski**	2014	1	avPM	2.0–8.0	5.2 ± 1.6	0.96	0.2154	− 0.8918
		2		1.0–7.0	3.3 ± 1.4	0.98	0.3913	− 0.4199
	2015	1	avPM	1.0–7.0	3.9 ± 1.4	0.95	0.1573	− 0.4424
**Modzurów**	2015	1	avPM	3.0–7.0	5.7 ± 0.9	0.98	− 0.8587	0.3902

**Table 2:** The Pearson’s correlation between mean values of powdery mildew resistance measured in 9-grade scale for all 168 DH lines of “Grenado” × “Zorro” mapping population evaluated in all localizations in three years (Ch, L, M—locations Choryń, Laski, and Modzurów respectively; 2013, 2014, 2015—season of experiments; 1, 2, 3—terms of observations)

	**Ch 2013_1**	**Ch 2013_2**	**Ch 2013_3**	**Ch 2014_1**	**Ch 2015_1**	**Ch 2015_2**	**Ch 2015_3**	**L 2014_1**	**L 2014_2**	**L 2015_1**
Ch 2013_2	0.5117 **									
Ch 2013_3	0.4165 **	0.8097 ***								
Ch 2014_1	− 0.0857	0.0013	− 0.0942							
Ch 2015_1	− 0.1969	-0.0642	− 0.0882	0.6687 **						
Ch 2015_2	− 0.1403	0.0142	− 0.0271	0.6772 **	0.9451 ***					
Ch 2015_3	− 0.2169 *	− 0.0277	− 0.0725	0.6236 **	0.8992 ***	0.9114 ***				
L 2014_1	0.0181	0.1149	0.0159	0.7870 ***	0.4849 **	0.5422 **	0.4970 **			
L 2014_2	− 0.1574	0.0687	0.0203	0.5787 **	0.5568 **	0.5848 **	0.6085 ***	0.5903**		
L 2015_1	− 0.0309	− 0.1757 *	− 0.2088 *	0.3550 *	0.5059 **	0.4698 **	0.4554 **	0.2052 *	0.2537 *	
M 2015_1	− 0.1110	− 0.2993 *	− 0.3308 *	0.3089 *	0.4912 **	0.4352 **	0.4770 **	0.0780	0.2680 *	0.6529 **

^*, **, ***^Significant at *P* < 0.05, *P* < 0.01 and *P* < 0.001, respectively.

### *The “Grenado”* × *“Zorro” linkage map*

A total of 1891 unique markers (1443 silicoDArT, 326 DArT, and 122 SNP) were assigned to 21 linkage groups corresponding to all triticale chromosomes (Table S1). However, for chromosomes 7A and 1B, additional separate linkage groups were discerned (7A.1 and 1B.1, respectively). These groups were left separate because combining them into a single linkage group was connected with the insertion of large gaps (above 30 cM). The genetic linkage map spanned 5249.9 cM with average marker density of 2.8 cM (Table 3). The A, B, and R genomes covered total distances of 1556.0, 1906.9, and 1787.0 cM, respectively. The A genome had the fewest markers assigned (538) and the highest markers saturation (3.0) comparing to the other triticale genomes. The total number of markers assigned the B and R genomes was 691 and 662, respectively with the corresponding maps saturation of 2.7 and 2.8 (Table 3).

**Table 3:** Summary of “Grenado” × “Zorro” linkage map containing silicoDArT, DArT, and SNP markers

Genome	Linkage group	Chrom. length (cM)	No. of markers				Markers saturation
			**SilicoDArT**	**DArT**	**SNP**	**All**	
A	1A	249.5	49	12	19	80	3.2
	2A	212.8	52	4	11	67	3.2
	3A	184.4	44	11	7	62	3.0
	4A	162.5	47	9	3	59	2.8
	5A	197.6	48	3	13	64	3.1
	6A	298.9	95	11	7	113	2.7
	7A	152.5	47	12	2	61	2.5
	7A.1	97.8	24	5	3	32	3.2
**A genome**	**8**	**1556.0**	**406**	**67**	**65**	**538**	**3.0**
B	1B	157.4	53	4	4	61	2.6
	1B.1	28.5	14	3	0	17	1.8
	2B	335.0	103	21	10	134	2.5
	3B	368.4	92	28	9	129	2.9
	4B	117.7	29	7	3	39	3.1
	5B	365.9	102	20	12	134	2.8
	6B	333.6	89	18	5	112	3.0
	7B	200.4	44	15	6	65	3.1
**B genome**	**8**	**1906.9**	**526**	**116**	**49**	**691**	**2.7**
R	1R	156.8	52	15	2	69	2.3
	2R	143.6	31	17	0	48	3.1
	3R	176.1	53	14	0	67	2.7
	4R	306.1	86	21	0	107	2.9
	5R	320.2	95	20	2	117	2.8
	6R	571.7	168	46	3	217	2.6
	7R	112.5	26	10	1	37	3.1
**R genome**	**7**	**1787.0**	**511**	**143**	**8**	**662**	**2.8**
**Total**	**23**	**5249.9**	**1443**	**326**	**122**	**1891**	**2.8**

### Detection of QTLs for powdery mildew resistance in triticale in all seasons and localizations

QTLs were calculated from the mean values of data obtained for each experiment separately. Identification of QTL associated with powdery mildew infection was carried out based on the genetic map created de novo for the “Grenado” × “Zorro” DH population. Composite interval mapping (CIM) identified total of 23 QTLs with LOD values ≥ 2.0 on 6 wheat (A and B) chromosomes: 4A, 7A, 7A.1, 2B, 3B, and 7B and 10 on rye (R) chromosomes: 1R, 4R, 5R, and 6R (Table 4, Fig. S2, Fig. S3).

**Table 4:** Characteristics of the quantitative traits loci associated with powdery mildew resistance in triticale located for AUDCP and avPM evaluated in all locations in all experimental years

QTL name	Flanking markers (position in cM)	LOD	LOD max. position (in cM)	Marker closest to the LOD peak	*R*^2^ (%)	Add	Favorable allele
**AUDCP Choryń 2013**							
***Qpm.gz.4R.1***	*3624369: 3614262* (60.5: 93.7)	6.8	61.1	*4372141*	15.2	207.82	G
		4.2	85.6	*4354376*	8.5	− 165.24	Z
***Qpm.gz.4R.2***	*3622032: 3608596* (161.9: 217.2)	2.4	170.9	*4200528*	4.5	42.95	G
***Qpm.gz.7A1.1***	*3046658: 4343552* (5.9: 29.8)	5.7	6.4	*4371107*	14.5	76.11	G
		6.8	18.9	*4358018*	16.2	79.88	G
**avPMChoryń 2014**							
***Qpm.gz.4R.3***	*rPt-400377: rPt-401230* (104.8: 116.4)	5.3	105.7	*rPt-401239*	13.6	− 0.46	G
***Qpm.gz.6R.1***	*4341045: rPt-506054* (55.8: 77.1)	3.4	67.0	*3608346*	7.9	-0.34	Z
***Qpm.gz.7A1.2***	*3046658: 4343525* (5.9: 29.8)	2.1	6.4	*4371107*	5.9	− 0.31	Z
***Qpm.gz.7B.1***	*4339655: 3606676* (162.5: 190.2)	2.3	177.6	*4344428*	5.3	− 0.33	Z
		3.5	181.4	*3623588*	8.6	− 0.39	Z
**avPM Laski 2014**							
***Qpm.gz.1R.1***	*4353991: 3609994* (67.3: 91.2)	3.9	70.1	*4342196*	6.2	0.43	G
		4.1	80.3	*3041555*	7.4	0.47	G
***Qpm.gz.4R.4***	*3612451: 4342913* (58.1: 68.9)	5.6	61.6	*4372141*	9.2	0.55	G
***Qpm.gz.4R.5***	*3622032: rPt-400365* (161.9: 189.7)	4.2	161.9	*3622032*	7.1	0.44	G
		5.5	175.2	*4347207*	8.8	0.49	G
***Qpm.gz.5R.1***	*4357257: 4218107* (95.2: 109.7)	2.8	115.5	*4206452*	4.4	− 0.43	Z
	*4357414: rPt-401500* (45.7: 60.5)	3.1	50.7	*3614922*	4.3	− 0.41	Z
	*4352431: 4348906* (0.0: 34.6)	3.0	21.8	*4349220*	4.2	− 0.38	Z
		3.0	2.0	*4348000*	4.2	− 0.40	Z
***Qpm.gz.7A1.3***	*4364739: wPt-0494* (69.4: 76.4)	8.3	70.3	*4350780*	14.4	0.64	G
**AUDCP Choryń 2015**							
***Qpm.gz.4R.6***	*3612451: 4342913* (58.1: 68.9)	5.2	65.0	*3610370*	11.7	− 196.2	Z
***Qpm.gz.6R.2***	*rPt-411293: 4354701* (203.2: 222.7)	3.3	203.2	*rPt-411293*	11.1	166.6	G
		3.0	215.5	*4341667*	6.4	137.2	G
***Qpm.gz.7A.1***	*4210062: 4221410* (33.0: 43.0)	2.7	34.0	*4344186*	5.8	− 141.3	Z
***Qpm.gz.7A1.4***	*wPt-6147: wPt-0745* (0.0: 16.3)	4.6	6.4	*4371107*	10.8	− 179.3	Z
***Qpm.gz.7B.2***	*4360157: 4220857* (179.5: 185.1)	6.5	181.4	*3623588*	14.9	216.2	G
**avPM Laski 2015**							
***Qpm.gz.2B.1***	*wPt-4072: 4366322* (308.9: 335.0)	2.6	308.9	*wPt-4072*	8.4	0.43	G
		3.0	326.2	*4357651*	7.7	0.43	G
***Qpm.gz.3B.1***	*3608740: wPt-1159* (93.2: 115.3)	2.1	94.5	*3610490*	5.7	− 0.35	Z
		2.5	105.5	*4344791*	5.9	− 0.36	Z
***Qpm.gz.7A1.5***	*3046658: 4343552* (5.9: 29.8)	4.8	18.9	*4358018*	15.2	0.60	G
**avPMModzurów 2015**							
***Qpm.gz.3B.2***	*3613639: 3609225* (104.0: 133.0)	3.0	115.3	*wPt-1159*	10.1	− 0.29	Z
***Qpm.gz.4A.1***	*4351892: 4343692* (90.1: 111.4	4.4	100.7	*4350881*	13.7	0.39	G
		3.4	107.5	*4373643*	11.5	0.33	G
***Qpm.gz.7B.3***	*4354063: 3606676* (174.3: 190.2)	4.5	177.6	*4344428*	14.8	− 0.41	Z
		5.4	181.4	*3623588*	17.3	− 0.43	Z
		3.3	185.1	*4220857*	11.7	− 0.34	Z

*Loci* associated with AUDPC evaluated in Choryń in 2013 and 2015 were located on chromosomes 7A, 7A.1, 4R, and 6R (Table 4, Fig. S2, Fig. S3). Those *loci* explained up to 15.2% and 16.2% of phenotypic variation for *Qpm.gz.4R.1* and *Qpm.gz.7A1.1* respectively. The highest LOD values were observed for *Qpm.gz.4R.1* (6.8), *Qpm.gz.7A1.1* (6.8 and 5.7), and *Qpm.gz.7B.2* (6.5, Table 4). Also, common QTL regions for both AUDPC measured in 2013 and 2015 were found on chromosomes 4R and 7A.1. *Locus Qpm.gz.4R.1* was co-located with *Qpm.gz.4R.6* on chromosome 4R between 60.5 cM and 68.9 cM as well as *Qpm.gz.7A1.1* with *Qpm.gz.7A1.4* on chromosome 7A.1 between 5.9 and 16.3 cM (Table 4, Fig. S2, Fig. S3).

The avPM which was measured within 2-year time period in three different locations revealed total of 15 *loci* associated with that trait on chromosomes 4A, 7A.1, 2B, 3B, 7B, 1R, 4R, 5R, and 6R (Table 4, Fig. S2, Fig. S3). Among of all 15 *loci*, the most significant QTLs are those stable over years and locations. On chromosome 7A.1, *loci Qpm.gz.7A1.2* and *Qpm.gz.7A1.5* were detected for avPM measured in Choryń location in 2014 and Laski in 2015 (Table 4, Fig. S2). These QTLs covered the same region on 7A.1 chromosome (5.9–29.8 cM) and explained 15.2% of phenotypic variation for *Qpm.gz.7A1.5* (Table 4). On chromosome 7B *loci*, *Qpm.gz.7B.1* and *Qpm.gz.7B.3* were detected between 174.3 and 190.2 cM in Choryń 2014 and Modzurów 2015 (Table 4, Fig. S2). It explained up to 17.3% of phenotypic variation and also, the same markers have peaked to the maximum LOD position (*4,344,428* and *3,623,588*). Additionally, on rye chromosome 5R one QTL *Qpm.gz.5R.1* was identified. This *locus* was composed of three regions—between 0.0 and 34.6 cM, 45.7–60.5 cM and 95.2–109.7 cM but all of them have a very similar additive effects and phenotypic variation (Table 4). Therefore, *Qpm.gz.5R.1* can be considered as one *locus* with effect split into three parts.

### Candidate genes for adult-plant resistance

Fourteen candidate genes were detected within 11 QTL regions identified in this study on chromosomes: 7A (3), 2B (1), 3B (2), 7B (2), 1R (1), 4R (1), 5R (3), and 6R (1) (Table 5). Among them, two gene records were repeated in different experiments. The first gene encoding GDSL esterase/lipase At4g28780-like (LOC119328445) was identified within *Qpm.gz.7A1.1*, *Qpm.gz.7A1.2*, and *Qpm.gz.7A1.5* found for AUDCP Choryń 2013, avPM Choryń 2014, and avPM Laski 2015 experiments. The second gene encoding CLAVATA3/ESR (CLE)-related protein 3-like (LOC119335261) was common for *Qpm.gz.4R.2* and *Qpm.gz.4R.5*. The four other candidate genes from QTLs *Qpm.gz.6R.1*, *Qpm.gz.1R.1*, *Qpm.gz.3B.2*, and *Qpm.gz.7B.3* coded different kinases like cyclin-dependent kinase A-2-like (LOC119314733), G-typelectin S-receptor-likeserine/threonine-protein kinase At2g19130 (LOC119294828), receptor-like protein kinase At3g47110 (LOC119266893) as well as serine/threonine-protein kinase-like protein ACR4 (LOC119325260), respectively. The remaining genes encoded: protein AMEIOTIC 1 homolog DYAD-like protein (LOC119308950), protein DETOXIFICATION 16-like (LOC119339835), putative disease resistance protein RGA3 (LOC119347815), sodium transporter HKT7-A1, uncharacterized F-box family protein (LOC109735658), uncharacterized ATP-dependent protease ATP asa subunit HslU (LOC119311530) as well as two uncharacterized proteins LOC109764755 and LOC113333611 (Table 5).

**Table 5:** Candidate genes for selected QTLs grouped by common/overlapping chromosome position

QTL name	Flanking markers (position in cM)	Candidate gene	Confidence	Position	Sequence ID	Predicted encoded protein	Predicted function
***Qpm.gz.7A1.1*** ***Qpm.gz.7A1.2*** ***Qpm.gz.7A1.5***	*3046658* *4343552* (5.9: 29.8)	*TraesCS7A03G1325200*	High	Chr7A:727065938.0.727068182 (− strand)	XM_037601429.1	*Triticumdicoccoides*GDSL esterase/lipase At4g28780-like (LOC119328445)	Extracellularhydrolase activity, acting on ester bonds; lipid catabolic process
		*TraesCS7A03G1253400*		Chr7A:706530491.0.706532316 (+ strand)	XM_020323539.2	*Aegilops tauschii* subsp. *strangulata* uncharacterized (LOC109764755)	–
***Qpm.gz.7A1.3***	*4364739: wPt-0494* (69.4: 76.4)	*TraesCS7A02G054900LC*	Low	Chr7A:20046305.0.20047051 (− strand)	XM_037616339.1	*Triticumdicoccoides* putative disease resistance protein RGA3 (LOC119347815)	ADP, ATP nucleic acid binding, zinc ion binding
***Qpm.gz.2B.1***	*wPt-4072: 4366322* (308.9: 335.0)	*TraesCS2B01G868700LC*	Low	Chr2B:772383880.0.772391111 (+ strand)	EF062820.1	*Triticummonococcum*putative sodium transporter HKT7-A1	Cation transmembrane transporter activity
***Qpm.gz.3B.1***	*3608740: wPt-1159* (93.2: 115.3)	*TraesCS3B02G143300LC*	Low	Chr3B:82447521.0.82449326 (− strand)	XM_026580037.1	*Papaver somniferum* uncharacterized (LOC113333611)	RNA binding (?)
***Qpm.gz.3B.2***	*3613639: 3609225* (104.0: 133.0)	*TraesCS3B02G089100LC*	Low	Chr3B:43818573.0.43818839 (+ strand)	XM_037548176.1	*Triticumdicoccoides* putative receptor-like protein kinase At3g47110 (LOC119266893)	ATP binding; protein serine kinase activity; protein threonine kinase activity
***Qpm.gz.7B.1***	*4339655: 3606676* (162.5: 190.2)	*TraesCS7B03G1287200*	High	Chr7B:745632916.0.745635602 (+ strand)	XM_037611732.1	*Triticumdicoccoides* protein DETOXIFICATION 16-like (LOC119339835)	Transmembrane antiporter activity; xenobiotic transmembrane transporter activity
***Qpm.gz.7B.3***	*4354063: 3606676* (174.3: 190.2)	*TraesCS6B02G067800*	High	Chr6B:45776705.0.45780939 (− strand)	XM_037598999.1	*Triticumdicoccoides* serine/threonine-protein kinase-like protein ACR4 (LOC119325260)	Membrane single-pass protein, endosomal protein; plant epidermal cell differentiation; protein autophosphorylation
***Qpm.gz.1R.1***	*4353991: 3609994* (67.3: 91.2)	*SECCE1Rv1G0001240*	High	Chr1R:4311740.0.4314268 (+ strand)	XM_037573103.1	*Triticumdicoccoides* G-type lectin S-receptor-like serine/threonine-protein kinase At2g19130 (LOC119294828)	ATP, calmodulin and carbohydrate binding; serine/threonine kinase activity; recognition of pollen
***Qpm.gz.4R.2*** ***Qpm.gz.4R.5***	*3622032: 3608596* (161.9: 217.2)	*SECCE4Rv1G0263150*	High	Chr4R:714644546.0.714644830 (− strand)	XM_037607406.1	*Triticumdicoccoides* CLAVATA3/ESR (CLE)-related protein 3-like(LOC119335261)	Extracellularreceptor serine/threonine kinase binding; cell–cell signaling involved in cell fate commitment
***Qpm.gz.5R.1***	*4357257: 4218107* (95.2: 109.7)	*SECCE5Rv1G0329130*	High	Chr5R:512707197.0.512708717 (+ strand)	XM_040389738.1	*Aegilops tauschii*subsp.*strangulata* uncharacterized (LOC109735658) F-box family protein	Plays a fundamental role in building the proper chromosome structure at the beginning of meiosis in malemeiocytes.
		*SECCE5Rv1G0299130*		Chr5R:14842927.0.14846132 (− strand)	XM_037585092.1	*Triticumdicoccoides* protein AMEIOTIC 1 homolog (LOC119308950)DYAD-like protein	Required for the transition from leptotene to zygotene in meiocytes. Required for homologouschromosomepairing
	*4357414: rPt-401500* (45.7: 60.5)	*SECCE5Rv1G0350710*	Low	Chr5R:691766950.0.691769067 (+ strand)	XM_037587158.1	*Triticumdicoccoides* uncharacterized (LOC119311530) ATP-dependent protease ATPase subunit HslU	ATP-dependent protease
	*4352431: 4348906* (0.0: 34.6)	*SECCE4Rv1G0234370*	High	Chr4R:277777386.0.277791698 (+ strand)	–	–	Beta-adaptin-like protein
***Qpm.gz.6R.1***	*4341045: rPt-506054* (55.8: 77.1)	*SECCEUnv1G0530270*	High	ChrUn:12044387.0.12047493 (− strand)	XM_037589426.1	*Triticumdicoccoides* cyclin-dependent kinase A-2-like (LOC119314733)	Negatively regulates endocycles and acts as a regulator of ploidy levels in endoreduplication. Promotes divisions in the guard cells (GCs) after the guard mother cells (GMC) symmetric division

## Discussion

Based on de novo mapping using unique silicoDArT, DArT, and SNP set of markers, the genetic map for triticale was constructed. This map was used to locate quantitative trait *loci* (QTL) associated with powdery mildew infection which was measured in a field conditions during 3-year period in three different locations across the Poland.

The genetic map created for “Grenado” × “Zorro” DH population was composed of 1891 markers assigned to 21 chromosomes which corresponds to triticale genome. The majority of this map was constructed of unique 1443 silicoDArT markers with 326 DArT and 122 SSR markers. DArT technique which is quick and highly reproducible can produce thousands of polymorphic *loci* in a single assay (Wenzl et al. 2004; Alam et al. 2018) that is why is widely used in genetic map construction for multiple crop species (Nsabiyera et al. 2020). However, DArT markers differ in intensity which may have an impact in some applications (Bolibok-Brągoszewska et al. 2009) that is why, a new genotyping technique, SNP chips has been developed and designed for a large number of SNPs (Nsabiyera et al. 2020; von Thaden et al. 2020). SNP chip method enables identification of quantitative trait loci (QTL) for different traits in various plant species (Ballesta et al. 2020; von Thaden et al. 2020). The total length of genetic map described in this paper was 5249.9 cM with the mean markers saturation 2.8 (3.0 for A, 2.7 for B, and 2.8 for R genome). Up to date, not many genetic maps were constructed and described for triticale (González et al. 2005; Alheit et al. 2011; Tyrka et al. 2011, 2015, 2018; Karbarz et al. 2020; Wąsek et al. 2021). The results of total marker number and mean map density are very similar to the genetic map of “Saka3006” × “Modus” DH mapping population described by Tyrka et al. (2011). From all markers, the highest number of them was assigned to the B genome (691) which is not corresponding to other described triticale genetic maps in contrast to the A genome with the lowest total number of markers (538). The A genome was previously described by Tyrka et al. (2011, 2015), Karbarz et al. (2020) and Wąsek et al. (2021) as the one with the lowest number of markers assigned, regardless of marker type used in map construction.

Based on the genetic map, detection of quantitative trait *loci* (QTL) associated with many important traits can be performed. Studies on localization of genomic regions in crops associated with resistance to fungal pathogens most often focused on fusarium head blight (Buerstmayr et al. 2002, 2003; Giancaspro et al. 2016; Clinesmith et al. 2019) and rusts (Melichar et al. 2008; Prins et al. 2011; Rosewarne et al. 2012; Li et al. 2020) especially in wheat. Regarding to powdery mildew resistance, identification of QTL was widely reported in wheat (Lan et al. 2010; Ren et al. 2017; Liu et al. 2020; Xu et al. 2020) in contrast to triticale (Karbarz et al. 2020). In this paper, detection of QTL regions linked to *B. graminis* resistance was tested in natural field conditions. Based on field results of triticale resistance, the area under disease progress curve (AUDPC) and the average value of powdery mildew infection (avPM) were calculated to obtain genomic regions associated with these traits.

On chromosome 4A, one *locus Qpm.gz.4A.1* was detected in observations conducted in Modzurów in 2015 that explained 13.7% of phenotypic variation (Table 4). On this chromosome, regions with high importance for wheat health were previously described (Chantret et al. 2001; Mingeot et al. 2002; Jakobson et al. 2012). Chromosome 4A has been reported a source of resistance genes not only to powdery mildew (*Pm16*) but also to leaf stripe and rust resistance (Reader and Miller 1991; Marone et al. 2012, 2013).

Six QTL regions were detected for both AUDPC and avPM in almost all experiments (except Modzurów location in 2015). Wheat chromosome 7A is known as a source of multiple *Pm* resistance genes (Yang et al. 2017;Nordestgaard et al. 2020) as well as QTL regions associated with powdery mildew resistance. Three of them, *Qpm.gz.7A1.1*, *Qpm.gz.7A1.2*, and *Qpm.gz.7A1.5* were found for AUDPC and avPM on the same position in a distance between 5.9 and 29.8 cM (Table 4, Fig. S2). Additionally, *locus Qpm.gz.7A1.4* was located between 0.0 and 16.3 cM for AUDPC with maximum LOD at the position of 6.4 cM (Table 4). Karbarz et al. (2020) reported *locus QPm-7A* in triticale associated with AUDPC of *B. graminis* infection in a distance between 0.0 and 23.3 cM which is very similar to results obtained in this study. Also, Chantret et al. (2001) described *loci* involved in adult-plant resistance (APR) on 7A in wheat *F*_2:3_ population which position of one of them coincides with *locus Qpm.gz.7A1.4*. Furthermore, the *Pm1* gene associated with the stem and leaf rust resistance genes *Sr15* and *Lr20* as well as gene *Pm37* are already reported on chromosome 7A (Neu et al. 2002; Marone et al. 2013). Additionally, genes associated with cellular hydrolase activity, acting on ester bonds, lipid catabolic process, ADP, ATP nucleic acid binding, and zinc ion binding were localized within QTLs on chromosome 7A (Table 5).

On chromosome arm 2BL, six powdery mildew resistance genes: *Pm6*, *Pm26*, *MlZec1*, *Pm33*, *MlLX9*, and *Pm51* were previously located (Zhan et al. 2014). In presented study, *locus Qpm.gz.2B.1* with LOD value 3.0 was found for avPM (Table 4) with a candidate gene TraesCS2B01G868700LC encoded cation transmembrane transporter activity (Table 5). Marone et al. (2013) also localized QTL region on this chromosome with marker *Xcdo244* corresponding to a *NBS-LRR* gene. Also, Asad et al. (2014) identified QTL for maximum disease severities (MDS) on this chromosome. *Locus QPm.caas-2BS.2* was mapped in a position which has a pleiotropic effect on both powdery mildew and stripe rust responses (Guo et al. 2008; Carter et al. 2009).

Two regions on chromosome 3B, *Qpm.gz.3B.1* and *Qpm.gz.3B.2* were found for avPM measured in 2015 in two different locations with a common chromosome region between 104.0 and 115.3 cM (Table 4, Fig. S2). The highest LOD value (3.0) and phenotypic variation (10.1%) were for *Qpm.gz.3B.2* with maximum LOD marker *wPt-1159* peak at 115.3 cM. Also, putative receptor-like protein kinase At3g47110 (LOC119266893) gene was located between 104.0 and 133.0 cM on this *locus* (Table 5). Two *loci* on a short and long arm of chromosome 3B were described by Asad et al. (2014) explained 9.1% and 18.1% of phenotypic variation. Both of those regions were in close location to *Pm13* and *Pm41* genes. Another *locus* on chromosome 3B was reported by Marone et al. (2013) with the marker *F103* peak on 3.9 cM position. Although, regions reported so far differ in a genetic position on 3B chromosome from QTL regions described in this paper, comparison of physical regions is necessary to suggest that both *loci* with high phenotypic variation effect can be a new source of powdery mildew resistance.

Three regions for both, AUDPC and avPM values from 2 years and two different locations were found on chromosome 7B. Those QTL have a common region in a distance from 174.3 to 185.1 cM with the highest LOD value (6.5) and phenotypic variation (14.9%) for *Qpm.gz.7B.2* (Table 4, Fig. S2). Genes in this region were involved in the transmembrane antiporter activity, xenobiotic transmembrane transporter activity, and plant epidermal cell differentiation (Table 5). Keller et al. (1999) identified *locus* on the position 134 cM to 158 cM in four out of the five environments. It was located on a long arm of this chromosome and linked to *Pm5* gene. Region described by Marone et al. (2013) was flanked by *wPt-8938* and *PmTm4* in a position of 137.7 cM on 7B. That *locus* can be confirmed by *Qpm.gz.7B.1* as this region starts from marker *4,339,655* in a position 162.5 cM which is in a close position to *wPt-8938* at 159.7 cM of “Grenado” × “Zorro” map (Tab. S1). Additionally, Chantret et al. (2001) and Mingeot et al. (2002) described *locus* on this chromosome associated with the resistance. These regions on 7B may correspond to *Qpm.gz.7B.1* to *Qpm.gz.7B.3*.

Localization of QTL regions and genes associated with powdery mildew resistance in rye is poorly described so far, comparing to wheat. But close relationship between wheat and rye allows the introduction of desirable agronomic traits from rye to wheat, such as tolerance to various abiotic factors, resistance to pests and fungal diseases, including resistance to powdery mildew (Crespo-Herrera et al. 2017). Long arm of 1R rye chromosome is widely used to obtain a new varieties of wheat using chromosomal translocation of 1BL.1RS or 1AL.1RS and transferring *Pm8* and *Pm17* genes into the wheat (Duan et al. 2017; Schlegel and Korzun 2021). Remaining rye chromosomes also contain genes which can be used to improve wheat cultivars (Landjeva et al. 2006). Genes *Pm7* and *Pm20*, from rye chromosomes 2RL and 6RL have been already transferred to many wheat cultivars causing powdery mildew resistance (Huang and Röder 2004; An et al. 2013, 2015; Guo et al. 2017; Schlegel and Korzun 2021).

In presented study, QTL regions for AUDCP and avPM have been identified on rye chromosomes 1R, 4R, 5R, and 6R (Table 4, Fig. S3). *Locus Qpm.gz.1R.1* on chromosome 1R, covered by markers in a distance 67.3 cM to 91.2 cM was detected for avPM in Laski in 2014. It explained up to 7.4% of phenotypic variation with the LOD value 4.1. The short arm of this chromosome is an important source of genes carrying resistance to leaf and stem rust, yellow rust, and powdery mildew (Schlegel and Meinel 1994; Landjeva et al. 2006) that may correspond to QTL region associated with powdery mildew resistance.

Total of six *loci* for both, AUDCP and avPM were found on chromosome 4R with the highest LOD value 6.8 and 15.2% of phenotypic variation for *Qpm.gz.4R.1*. For those, two common regions were identified on a distance 60.5–68.9 cM and 161.9–189.7 cM (Table 4, Fig. S3). Within all identified *loci* on 4R, CLAVATA3/ESR (CLE)-related protein 3-like protein was found in SECCE4Rv1G0263150 candidate gene (Table 5). It has been reported that rye chromosome 4R contains the elite pool of genes which are applicable for wheat cultivar improvement (Duan et al. 2017). Up to date, five *Pm* genes derived from rye have been identified and transferred into the wheat genome, especially *Pm8* which is one of the most effective and has made a contribution to control wheat powdery mildew (Huang and Röder 2004; Ma et al. 2020). Additionally, Karbarz et al. (2020) described a *locus* on 4R triticale chromosome, detected for AUDPC which flanking marker *rPt-505620* in a position of 175.2 cM is in a close distance to flanking marker *rPt-401230* of *Qpm.gz.4R.3* at 116.4 cM. We can infer that two new resistance loci to powdery mildew corresponding to three QTLs common with *Qpm.gz.4R.1* and two QTLs from region of *Qpm.gz.4R.2* were identified.

*Qpm.gz.5R.1* region, identified for avPM in Laski in 2014 consisted of three regions separated from each other by 11.1 cM and 34.7 cM (Table 4, Tab. S1). But due to very similar phenotypic and additive effects, it has been considered as one *locus* on 5R chromosome. Most of the genes located within QTL on chromosome 5R were involved in building the proper chromosome structure at the beginning of meiosis, transition from leptotene to zygotene and homologous chromosome pairing (Table 5). These genes can potentially be important for maintaining the proper functioning of the plant genome despite the ongoing stress associated with powdery mildew infection and defense processes. No QTL for powdery mildew has been detected on the 5R rye chromosome to date so it might be reported as a new source of resistance. To make this effect stronger, the existence of *Pm4* gene on this chromosome was confirmed as well as a genes controlling resistance to leaf rust (Baranova et al. 2002; Tyrka and Chelkowski 2004).

Two regions on 6R chromosome were detected for AUDCP and avPM in Choryń in a 2-years period (2014 and 2015). Those *loci* were in a different position on this chromosome and explained up to 11.1% of phenotypic variation for *Qpm.gz.6R.2* and LOD value 3.4 for *Qpm.gz.6R.1*. Also, for *Qpm.gz.6R.1*, gene encoded cyclin-dependent kinase A-2-like (LOC119314733) protein was identified (Table 5). The *Pm20* gene has been identified and derived from 6RL of Prolific rye (Zhuang 2003; An et al. 2015) that may correspond to one QTL region on the 6R rye chromosome associated with powdery mildew resistance, while the second *locus* on this chromosome is new.

In conclusion, availability of the winter triticale DH population allowed to create a new, high-density genetic map for this crop specie. Based on this map, a total of 23 QTL regions were identified based on a 3-year field experiment on triticale resistance to powdery mildew infection conducted in three different locations across the Poland. Among those regions, two found on rye chromosome 4R and single *loci* on 5R and 6R were reported for the first time as regions associated with powdery mildew resistance. The information of significant QTL regions associated with powdery mildew resistance together with candidate gene–coded proteins taking part in triticale defense against fungal pathogen can be an important tool used in modern breeding programs. Molecular markers against *Blumeria graminis* after careful validation in available triticale varieties can be used for pyramiding two or more than two APR genes or QTLs from donor to recipient parent. To assist molecular breeding programs, described in this paper, regions associated with PM resistance can be used in marker-assisted selection (MAS) as well as in marker-assisted recurrent selection (MARS) and genomic selection (GS).

## Supplementary Information

Below is the link to the electronic supplementary material.

Supplementary file1 (PDF 76 KB)

Supplementary file2 (PDF 7783 KB)

Supplementary file3 (PDF 7693 KB)

Supplementary file4 (XLSX 208 KB)
