# Supplementary figures and images for: Genetic mapping of adult-plant resistance genes to powdery mildew in triticale
Source: J Appl Genet. 2021 Sep 24;63(1):73–86. doi: 10.1007/s13353-021-00664-x (PMC8755695; doi:10.1007/s13353-021-00664-x)

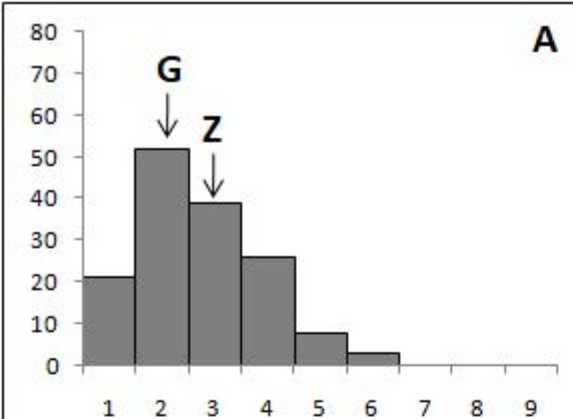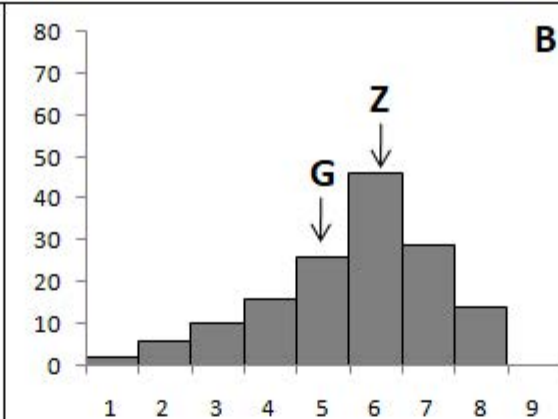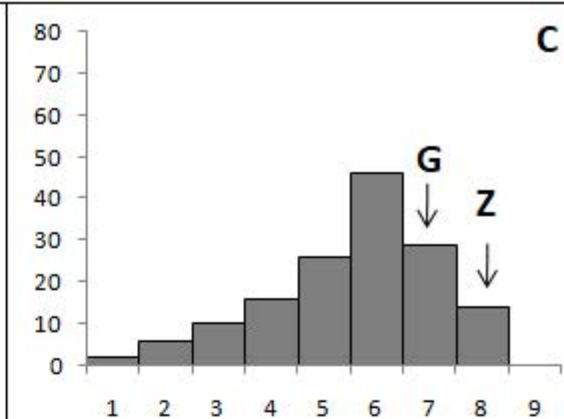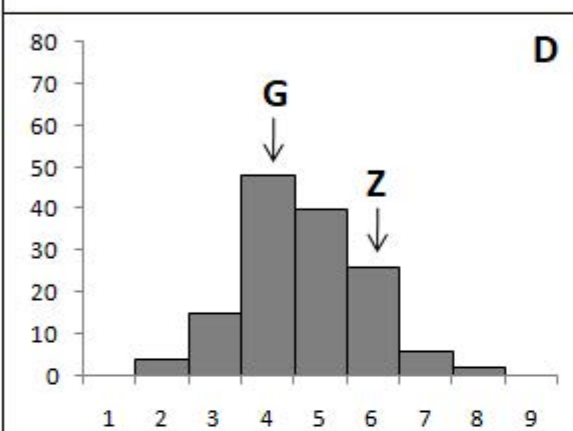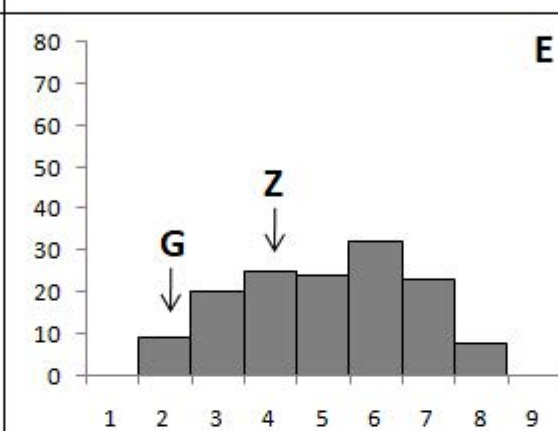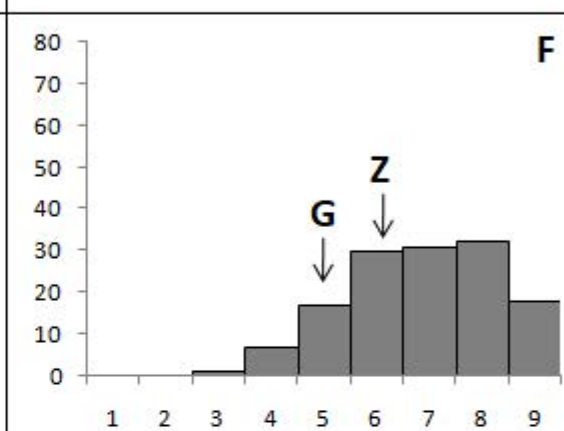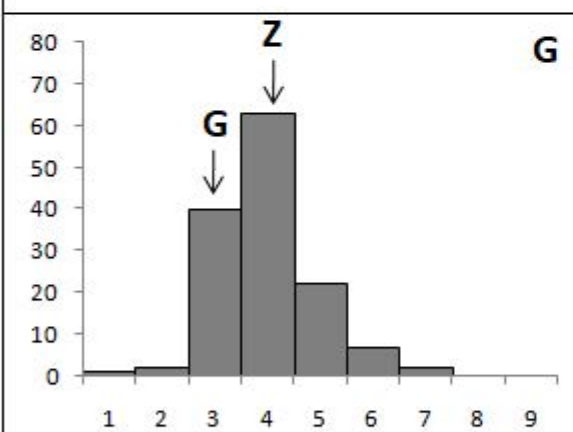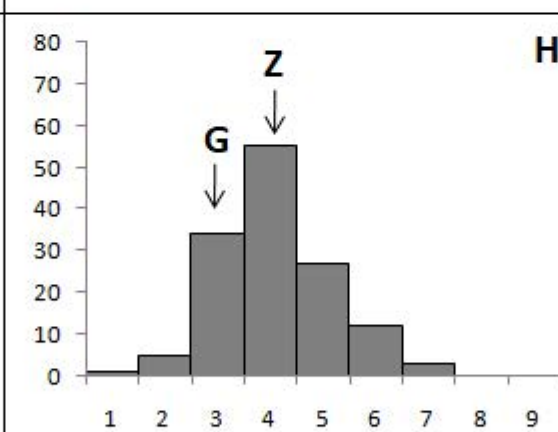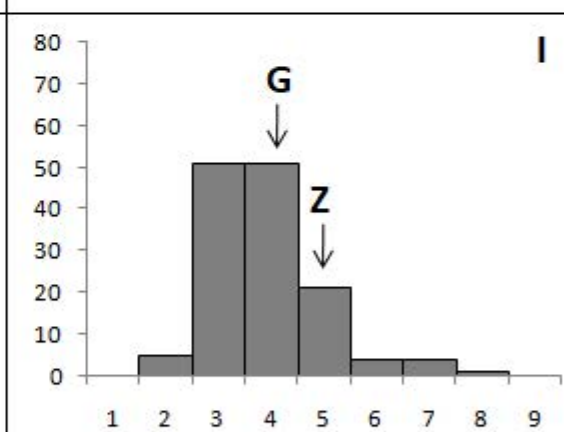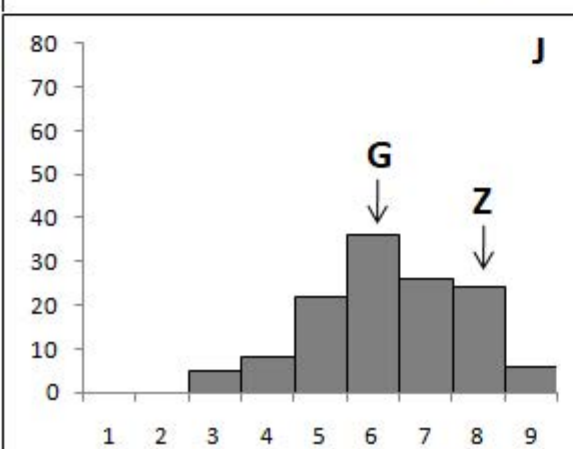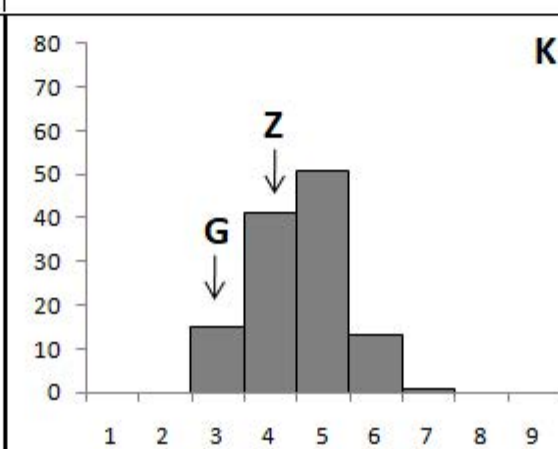

Supplement: Supplementary file 1 — Supplementary file1 (PDF 76 KB) [file 13353_2021_664_MOESM1_ESM.pdf]

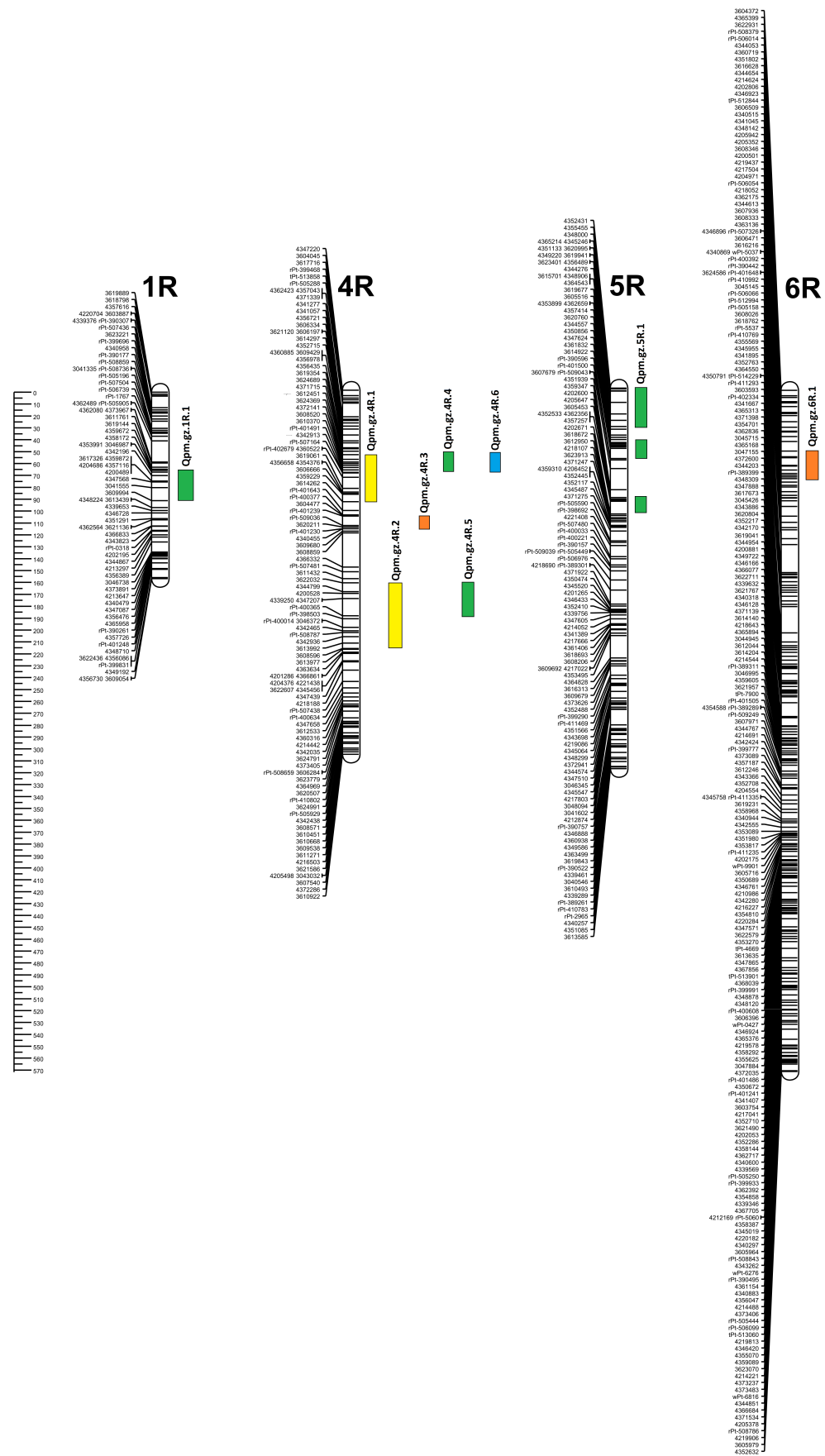

Supplement: Supplementary file 2 — Supplementary file2 (PDF 7783 KB) [file 13353_2021_664_MOESM2_ESM.pdf]

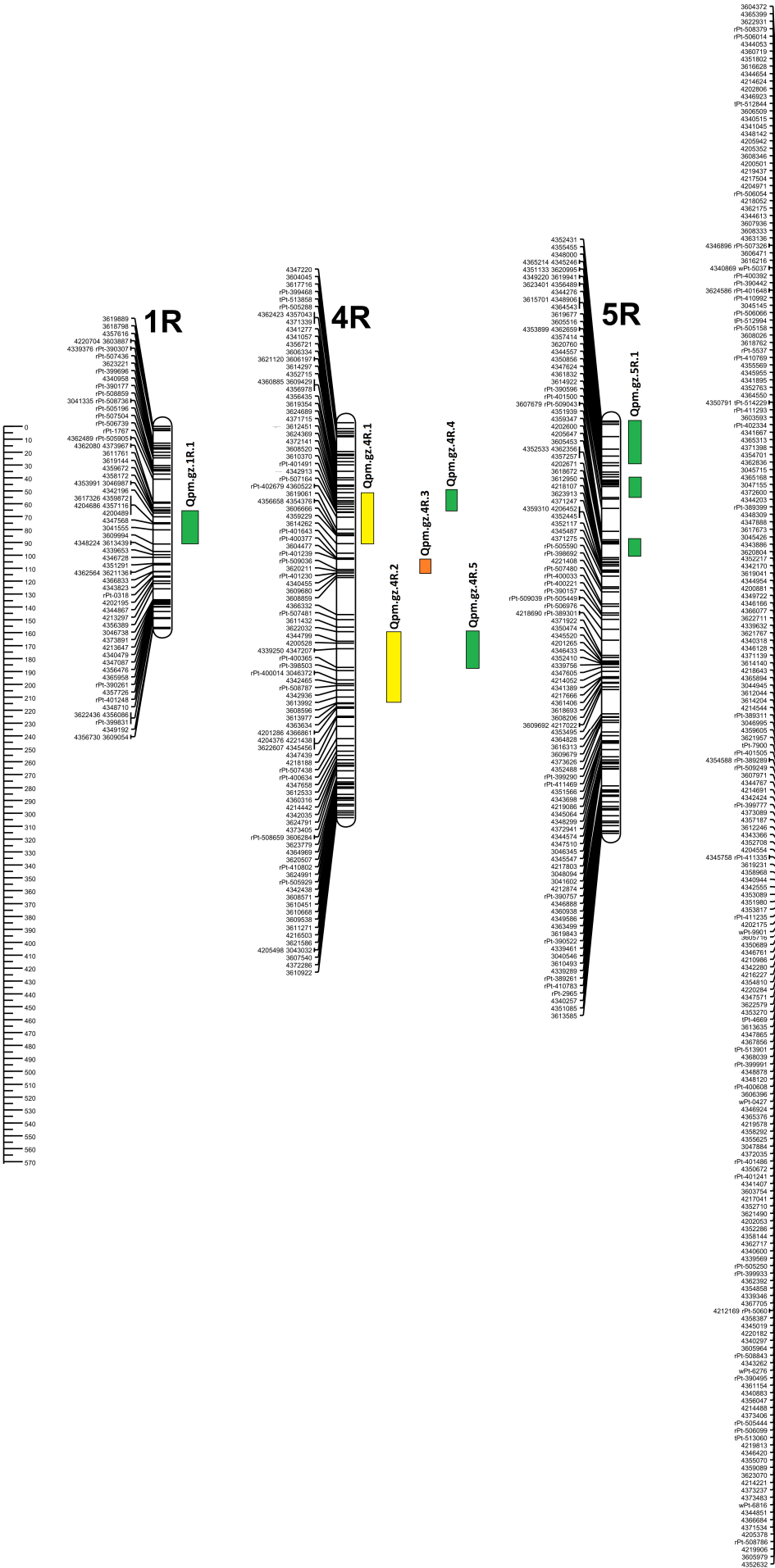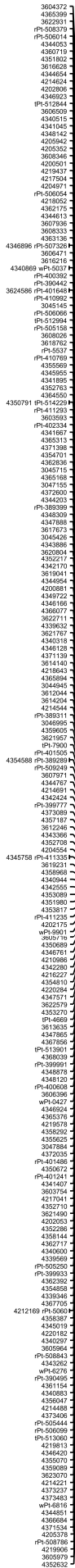

Supplement: Supplementary file 3 — Supplementary file3 (PDF 7693 KB) [file 13353_2021_664_MOESM3_ESM.pdf]
